# Validation of a questionnaire to monitor symptoms in HIV-infected patients during hepatitis C treatment

**DOI:** 10.1186/s12981-017-0182-7

**Published:** 2017-09-20

**Authors:** Edward R. Cachay, Craig Ballard, Bradford Colwell, Francesca Torriani, Charles Hicks, Wm. Christopher Mathews

**Affiliations:** 1 Department of Medicine-Owen Clinic, University of California at San Diego, 200 W. Arbor Drive, San Diego, CA 92103-8681 USA; 2 Division of Infectious Diseases, University of California at San Diego, 9500 Gilman Drive #0711, La Jolla, CA 92093-0711 USA; 3 Skaggs School of Pharmacy and Pharmaceutical Sciences, UC San Diego, 9500 Gilman Drive, La Jolla, CA 92093-0021 USA

**Keywords:** HIV, HCV treatment, Symptoms, Monitoring

## Abstract

**Background:**

Clinicians are incorporating patient-reported outcomes in the management of HIV-infected persons co-infected with hepatitis C virus (HCV), but there are no validated inventories to monitor symptoms of patients during HCV therapy.

**Design:**

Five-year retrospective cohort analysis of persons living with HIV (PLWH) treated for HCV.

**Methods:**

The HCV symptom-inventory (HCV-SI) was administered before, during, and after HCV treatment. Discriminant validity was assessed, separately, in mixed model linear regression of HCV-SI T-scores on treatment regimens (pegylated-interferon and ribavirin; pegylated-interferon, ribavirin, and telaprevir; and interferon-free antivirals); and side effect-related premature treatment discontinuation (SE-DC).

**Results:**

From the 103 patients who completed the HCV-SI, 7% were female, 26% non-white, 32% cirrhotics and 91% had undetectable HIV viral loads. Most had genotype 1 (83%) and were HCV treatment-naïve (78%). We treated 19% of patients with pegylated-interferon and ribavirin, 22% with pegylated-interferon, ribavirin, and telaprevir and 59% received interferon-free antivirals. Overall, 77% achieved a sustained virologic response, and 6% discontinued HCV treatment due to side effects. In the treatment discrimination model, compared to the no treatment period, HCV-SI scores were significantly (p < 0.01) lower for interferon-free antivirals and higher for interferon-containing regimens. In the SE-DC model, the total HCV-SI, somatic and neuropsychiatric scores significantly predicted those patients who prematurely discontinued HCV treatment (P < 0.05).

**Conclusions:**

The HCV-SI effectively differentiated among treatment regimens known to vary by side effect profiles and between patients with and without treatment discontinuation due to side effects. The HCV-SI may have value as a patient-reported outcome instrument predicting the risk of HCV treatment discontinuation.

**Electronic supplementary material:**

The online version of this article (doi:10.1186/s12981-017-0182-7) contains supplementary material, which is available to authorized users.

## Background

Direct-acting antivirals (DAA) provide safe and curative treatment for hepatitis C virus (HCV) in patients who start and complete therapy successfully [1].

Clinicians treating HCV are increasingly incorporating patient-reported outcomes in clinical trials and routine care settings to better understand the interaction of complex patient-related factors [2]. Although clinical trials have used instruments that mainly focus on disease-specific health quality of life such as The Chronic Liver Disease Questionnaire-Hepatitis C Virus [3], there are no standard quantitative monitoring tools that can systematically track different symptom domains of patients while on HCV therapy. Assessing symptoms of persons living with HIV (PLWH) while undergoing HCV-related health management is important as many PLWH may still present with non-specific psychosomatic symptoms before providers recognize potentially causative issues. These potential issues include relapse of substance/alcohol with resultant worsening depression, low perceived self-esteem, and/or poor adherence to HCV or antiretroviral medications [4]. PLWH have a high prevalence of medical comorbidities and ongoing barriers to care such as substance/alcohol use, unstable housing, and neuropsychiatric disease [5]. These factors can unfavorably impact patient medication tolerance, perception and willingness to complete HCV therapy, even when treating their HCV with shorter, better-tolerated, interferon (IFN)-free DAA [6, 7].

We previously validated a 41-item HCV symptom inventory (HCV-SI) as a predictor of HCV treatment initiation using pegylated interferon and ribavirin in PLWH. The inventory consists of a clinically meaningful, three-factor structure that includes neuropsychiatric, somatic, and sleep symptoms. The three-factor subscales demonstrated excellent internal consistency, reliability, and predictive validity [8].

The composition of HCV treatment regimens can serve as a validation criterion for an HCV symptom inventory, based on findings from clinical trials that symptom burden reflects the components of the HCV regimen. Therefore, we conducted the present study to compare the potential of a multidimensional HCV-SI score: (1) to discriminate between IFN-containing and IFN-free treatment regimens; and (2) to identify patients with premature treatment discontinuation and/or loss to follow-up due to worsening symptoms or side effects.

## Methods

The Owen Hepatitis Co-Infection Clinic was founded in April 2008 as a multidisciplinary HCV primary care-based program at the University of California San Diego (UCSD) Medical Center. Since its inception, it has used inclusive protocols aimed at increasing HCV treatment uptake among PLWH, including those with ongoing drug use, alcohol consumption, neuropsychiatric disease, and unstable housing while fulfilling our minimum eligibility criteria [9]. In short, the pre-treatment assessment for HCV treatment eligibility among patients with ongoing barriers to care required only: (1) consistent undetectable HIV viral load, (2) stable concurrent medical comorbidities, (3) favorable assessment by the team’s psychiatrist when there is a history of a psychiatric condition that may interfere with treatment, (4) encouraging registration with a needle exchange program for those with ongoing injection drug use, and (5) alcohol sobriety or controlled drinking for at least one month before HCV treatment initiation [9]. To further investigate the psychometric properties of the HCV-SI, we assembled a retrospective longitudinal cohort of adult PLWH treated for HCV between December 2011 and May 2016.

The HCV-SI is incorporated in the substance, alcohol and depression screening as part of our standard of care every time a patient attends the Owen HCV Hepatitis Co-Infection Clinic. The testing is conducted before, during, and after HCV treatment. The first page of the evaluation contains a standard validated depression inventory (PHQ-9/CES-D), the second page includes the NIDA-ASSIST for illegal substance use and AUDIT-C for alcohol abuse assessment, and the third page contains the HCV-SI questions (Additional file 1: Table S1). We use the online survey platform SurveyGizmo for the HCV-SI. Regarding the HCV-SI, each symptom item includes a 5-category, Likert-scaled symptom severity response, ranging from 0 (symptom absent) to 4 (symptom very severe). The total HCV-SI score could range from 0 to 164. As previously reported [4], the 41-item HCV-SI yielded a three-factor structure explaining 60% of the variance for the inventory. Factor 1 (neuropsychiatric symptoms) had 17 items, factor 2 (somatic symptoms) had eleven items, and factor 3 (sleep symptoms) had two items, explaining 28, 22 and 11% of the variance, respectively. Internal consistency reliability estimates of the three subscales were 0.93, 0.89 and 0.79 for neuropsychiatric, somatic and sleep symptoms, respectively. The possible ranges of observable raw scores for the HCV-SI and its subscales were: 0–164 (HCV-SI total), 0–44 (somatic), 0–8 (sleep), and 0–68 (neuropsychiatric). We present raw sum scores, T-scores, and POMP-scores (percent of maximum possible) [10].

Per our HCV clinic protocol, following HCV therapy initiation patients are assessed monthly for the duration of the treatment. After treatment completion, patients returned after 1, 2 and 6 months during the INF-based era and, in the IFN-free DAA era, they returned after 1 and 3 months for SVR ascertainment. The HCV-SI is self-administered by patients prior to their clinic visit, taking approximately 5 min to complete. The last time that HCV-SI was administered in any given patient was during the clinical visit for SVR ascertainment. Patients who completed the HCV-SI during at least two phases of HCV treatment (before, during, or after treatment) were included in the analysis. Demographic data, substance use, psychiatric history, and comorbidity data were obtained from medical records.

Total HCV-SI scores were examined in longitudinal scatter plots with linear and lowess smoothing to select the distribution that best fits study data. Discriminant validity was assessed separately for criteria treatment regimen composition and premature treatment discontinuation, in a mixed model linear regression that accounts for repeated measures of the HCV-SI T-scores. Raw summated total HCV-SI and subscale scores were transformed to T-scores (with mean 50 and standard deviation 10) to facilitate comparison of regression model coefficients across models varying by SI total or subscale dependent variables [11]. Our analyses focused on four dependent variables (T-scores for the total HCV-SI and the somatic, sleep, and neuropsychiatric subscales) and two independent discrimination variables (regimen composition and premature treatment discontinuation). To examine all selected variables, we fit by maximum likelihood separate mixed model random intercept linear regressions with robust standard errors using xtmixed (StataCorp. 2016. Stata Statistical Software: Release 14.2. College Station, TX).

Examination of discriminant validity of the HCV-SI was conducted using mixed model regression to compare HCV-SI scores while patients were receiving HCV treatment in the three-different groups, as follows: patients treated with pegylated interferon/ribavirin; those treated with pegylated interferon/ribavirin/telaprevir; and those treated with IFN-free DAA regimens. We estimated the treatment regimen effects using the non-treatment periods as the comparison reference category. For the premature treatment discontinuation criterion, we dichotomized patients into two groups according to whether they completed (reference category) or prematurely discontinued treatment due to side effects or loss to follow-up. We reported the intraclass correlation coefficient (ICC) for each model as a measure of the proportion of total variance in the dependent variable accounted for by clustering within subjects [12].

We conducted Harrell’s c-statistics, equivalent to the receiver operating characteristics (ROC) area, to evaluate the ability of the HCV-SI and its subscales to discriminate between the levels of the two discriminating criteria. The Harrell’s c-statistic was estimated for binary specifications of the criteria while taking into account within-subject clustering for (1) treatment regimen composition (combining both interferon-containing regimens in comparison with the combined no treatment and interferon-free DAA arms [reference category]) and (2) premature treatment continuation. The c-statistic was estimated using the Stata function somersd, which decomposes the ROC area into between and within cluster components [13]. Finally, we compared the discrimination (Harrell’s c) of the total HCV Symptom Inventory T-score for the two discriminating outcomes to the comparable discrimination of PROMIS depression T-scores ascertained at the same clinic visits. Because both PHQ-9 and CES-D depression instruments were utilized during the study, we employed linking tables to transform both PHQ-9 and CES-D depression scores to PROMIS depression T-scores that were used in the analysis [14].

## Results

During the study period, we treated 156 PLWH for HCV. Two patients (1.3%) were not eligible to complete the HCV-SI due language barriers. Of the remaining 154, 51 were excluded [five (3.2%) declined to participate, and 46 (29.5%) had fewer than two-time phases completed HCV-SI)] leaving 103 to comprise the study population. There were no differences in demographic characteristics or prevalence of barriers to care (drug or alcohol use, active psychiatric disease or unstable housing), HIV risk factors, CD4 cell count, HIV viral suppression, HCV genotype distribution, cirrhosis diagnosis, prior liver decompensation or prior HCV treatment history outcomes between those patients included versus those excluded from the study (Additional file 2: Table S2). Among the 46 patients who completed the HCV-SI in only one-time phase and were excluded from the main analysis, 25 had documented reasons for lack of completion such as a new competing medical priority (12), failure to return for follow-up and required outreach efforts to ascertain their HCV treatment outcome (5), relocated soon after starting HCV therapy (4), and incarceration (4). Likely patient fatigue was the main reason in 45.6% of patients who completed only one time-phase the HCV-SI (21 of 46). Table 1 shows the demographic, clinical, and HIV characteristics of the study population which were mostly male (93%), white (74%), and had a history of intravenous drug use (IDU—70%). Most patients had HCV genotype 1 infections and were naïve to HCV treatment. Overall, 32% of included patients had cirrhosis. Intolerance to previous HCV treatment was rare (6%). At the time of first HCV-SI measurement, 91% of patients had a suppressed HIV viral load.

**Table 1:** Demographic, HCV and HUV related characteristics of study patients

Factor	Total cohort (n = 103)	Peg-IFN + RBV (n = 19)	Peg-IFN + RBV + TPV (n = 23)	IFN-free DAA (n = 61)	P value*
Age in years, mean (SD)	49.7 (9.3)	43.6 (12.1)	45.3 (7.8)	53.2 (7.0)	< 0.001
Sex					
Female	7 (6.8%)	2 (11%)	3 (13%)	2 (3%)	0.17
Male	96 (93.2%)	17 (89%)	20 (87%)	59 (97%)	
Race					
Non-white	27 (26.2%)	5 (26%)	6 (26%)	16 (26%)	1.00
White	76 (73.8%)	14 (74%)	17 (74%)	45 (74%)	
Ethnicity					
Not hispanic	85 (82.5%)	14 (74%)	17 (74%)	54 (89%)	0.14
Hispanic	18 (17.5%)	5 (26%)	6 (26%)	7 (11%)	
HIV risk factor					
MSM	24 (23.3%)	4 (21%)	3 (13%)	17 (28%)	0.55
Heterosexual	1 (1.0%)	0 (0%)	0 (0%)	1 (2%)	
Hemophilia	6 (5.8%)	0 (0%)	3 (13%)	3 (5%)	
MSM + IDU	45 (43.7%)	11 (58%)	11 (48%)	23 (38%)	
Heterosexual IDU	27 (26.2%)	4 (21%)	6 (26%)	17 (28%)	
CD4 + in cells/mm^3^, median (IQR)	476.0 (341, 693)	607.0 (419, 719)	584.0 (426, 772)	417 (306, 598)	0.045
HIV viral load in copies/mL					
≤ 50	94 (91.3%)	17 (89%)	20 (87%)	59 (97%)	1.00
> 50	9 (8.7%)	5 (26%)	6 (26%)	16 (26%)	
HCV viral load in millions IU/L, median (IQR)	2.0 (0.4, 5.8)	3.8 (0.3, 9.7)	3.6 (1.2, 17)	1.3 (0.4, 3.5)	0.017
HCV genotype					
1/1A/1B	86 (83.5%)	13 (68%)	23 (100%)	50 (83%)	0.014
2	4 (3.8%)	1 (5%)	0 (0%)	3 (5%)	
3	11 (10.7%)	5 (26%)	0 (0%)	6 (10%)	
4	2 (1.9%)	0 (0%)	0 (0%)	2 (3%)	
HCV treatment history					
Naïve	80 (77.7%)	19 (100%)	16 (70%)	45 (74%)	0.19
IFN-intolerant	6 (5.8%)	0 (0%)	1 (4%)	5 (8%)	
Relapser	10 (9.7%)	0 (0%)	5 (22%)	5 (8%)	
Null responder	6 (5.8%)	0 (0%)	1 (4%)	5 (8%)	
Cure but reinfected	1 (1.0%)	0 (0%)	0 (0%)	1 (2%)	
Cirrhosis status					
Non-cirrhotic	70 (68.0%)	17 (89%)	17 (74%)	36 (59%)	0.032
Cirrhotic	33 (32.0%)	2 (11%)	6 (26%)	25 (41%)	
Prior decompensation or CPS > B					
Compensated cirrhosis	20 (19.4%)	2 (11%)	4 (17%)	14 (23%)	0.13
Prior decompensated cirrhosis	13 (12.6%)	0 (0%)	2 (9%)	11 (18%)	
Non-cirrhotic	70 (68.0%)	17 (89%)	17 (74%)	36 (59%)	
SVR on treatment 1					
Yes	78 (76.5%)	12 (63%)	13 (57%)	53 (88%)	1.00
No, null response	7 (6.9%)	2 (11%)	5 (22%)	0 (0%)	
No, relapse	4 (3.9%)	3 (16%)	0 (0%)	1 (2%)	
No, sides effects/adverse events	9 (8.8%)	2 (11%)	5 (22%)	2 (3%)	
No, died during treatment	4 (3.9%)	0 (0%)	0 (0%)	4 (7%)	
Discontinuation due to severe adverse events (1st regimen)					
No	93 (91.2%)	17 (89%)	18 (78%)	58 (97%)	0.021
Yes	9 (8.8%)	2 (11%)	5 (22%)	2 (3%)	
No. HCV-SI observations, median (IQR)	7.0 (4.0, 12.0)	8.0 (6.0, 15.0)	15.0 (11.0, 20.0	5.0 (4.0, 7.0)	< 0.001
Barriers to care					
Ongoing drugs/alcohol use	30 (29%)	8 (42%)	7 (30%)	15 (25%)	0.34
Active psychiatric disease	28 (27%)	8 (42%)	6 (26%)	14 (23%)	0.26
Unstable housing	5 (5%)	1 (5%)	3 (13%)	1 (2%)	0.10

*Peg*-*IFN* Pegylated interferon, *IFN* Interferon, *RBV* Ribavirin, *DAA* Direct acting antiviral, *MSM* Men who have sex with men, *IDU* Intravenous drug use, *HCV* Hepatitis C virus, *SVR* Sustained viral response, *CPS* Child–Pugh score, *SD* Standard deviation, *IQR* Interquartile range, *HCV*-*SI* Hepatitis C symptom inventory

* P value for comparison of the 3 groups: Peg-IFN + RBV vs. Peg-IFN + RBV + TPV vs. IFN-Free DAA

By HCV treatment group, 19% (n = 19) of study patients were treated with pegylated interferon/ribavirin, 22% (n = 23) with pegylated-interferon/ribavirin/telaprevir, and 59% (n = 61) with IFN-free DAA. The IFN-free DAA regimens included sofosbuvir/ledipasvir (n = 39), sofosbuvir/simeprevir (n = 11), sofosbuvir/ribavirin (n = 8), sofosbuvir/daclatasvir (n = 1) and two patients received ombitasvir/paritaprevir/ritonavir plus dasabuvir and ribavirin. There were no differences between treatment groups in gender, race/ethnicity, HIV risk factor, the proportion of patients with suppressed HIV viremia and prior HCV treatment history outcomes (Table 1). Overall, 77% of patients achieved an HCV sustained viral response (SVR). Patients treated with IFN-free DAA achieved the highest SVR (88%) despite the high proportion of patients with cirrhosis in this group. Patients treated with pegylated interferon/ribavirin and pegylated interferon/ribavirin/telaprevir achieved an HCV SVR in 63 and 57%, respectively.

**Fig. 1:**
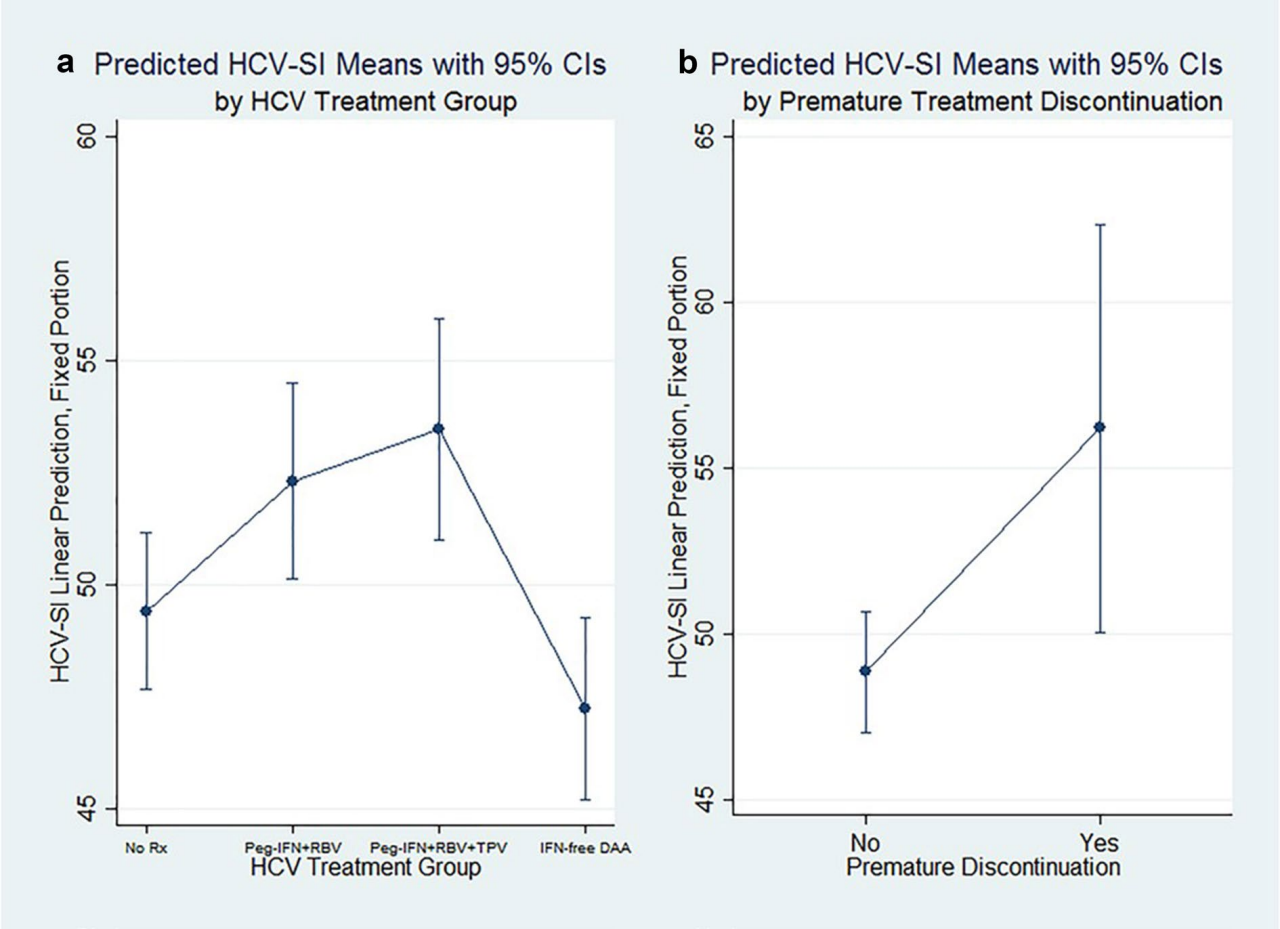
**a** Shows plots with 95% confidence interval of the average T-scores of the symptoms of each treatment group while undergoing HCV therapy. **b** Illustrates plots with 95% confidence intervals of the symptom scores when patients were dichotomized based on their hepatitis C treatment premature discontinuation status due to adverse events or lost to follow-up

Table 2 presents the HCV-SI total and subscale score distributions of 868 responses from 103 patients. The median number of times that the HCV-SI was completed by each patient while on HCV therapy was 7 (range 4–12). Patients receiving IFN-containing regimens developed worsening neuropsychiatric and somatic symptoms (with stable sleep symptoms) compared to those who did not receive HCV treatment. In contrast, those treated with IFN-free DAA had significantly fewer reported symptoms than those who did not receive HCV treatment (Table 2). The estimated coefficient beta (b) of the models presented in Table 3 may be interpreted using the following example for the total HCV-SI T-score as the dependent variable. For patients not on HCV therapy (reference group), the average total symptom score was 49.42. For patients treated with IFN-free DAA, the average total symptom score was 47.24 (49.42–2.18), while the corresponding scores for patients treated with pegylated interferon/ribavirin/telaprevir, and pegylated interferon/ribavirin were 53.48 (49.42 + 4.06) and 52.31 (49.42 + 2.89), respectively. Figure 1a, depicts the findings of the treatment discrimination model, compared to no treatment period, where the predicted total HCV-SI marginal scores (fixed portion) of patients treated with IFN-free DAA are significantly lower than those treated with IFN-containing regimens (p < 0.001).

**Table 2:** Distribution of HCV-SI total and subscale scores (raw, T-scores, and percent of maximum possible scores)

Scale	Mean (SD)
Number of patients	103
Number of responses	868
HCV-SI all symptoms [raw]	26.9 (24.0)
T-score, all symptoms	50.0 (10.0)
HCV-SI total, POMP	16.4 (14.6)
Neuropsychiatric symptoms [raw]	10.1 (9.8)
T-Score, neuropsychiatric	50.0 (10.0)
Neuropsychiatric, POMP	14.9 (14.4)
Somatic symptoms [raw]	7.2 (7.9)
T-score, somatic	50.0 (10.0)
Somatic, POMP	16.5 (18.0)
Sleep symptoms [raw]	2.3 (2.4)
T-Score, sleep	50.0 (10.0)
Sleep, POMP	29.3 (30.1)

*SD* standard deviation, *HCV-SI* hepatitis c symptom inventory, POPM percent of maximum possible scores

**Table 3:** Mixed model regression: symptom (subscales) T-scores on treatment category

Treatment group (Reference: no treatment)	T-score, all symptoms B (95% CI)	T-score, somatic B (95% CI)	T-score, sleep B (95% CI)	T-score, neuropsychiatric B (95% CI)
Peg-IFN + RBV	2.89 (1.03, 4.75)**	2.36 (0.33, 4.38)*	1.85 (−0.21, 3.91)	2.43 (0.48, 4.39)*
Peg-IFN + RBV + TPV	4.06 (2.23, 5.89)***	4.22 (2.54, 5.89)***	0.59 (−1.68, 2.85)	2.88 (0.94, 4.82)**
IFN-free DAA	−2.18 (−3.78, − 0.58)**	−1.763 (−3.22, − 0.31)*	−0.99 (−2.53, − 0.55)	−2.23 (−3.95, − 0.52)*
Constant	49.42 (47.69, 51.15)***	49.29 (47.64, 50.95)***	49.93 (48.35, 51.52)***	49.95 (48.16, 51.75)***
No. of patients	103	103	103	103
ICC	0.62	0.63	0.60	0.59

*B* Beta coefficient, *CI* Confidence intervals, *Peg*-*IFN* Pegylated interferon, *RBV* Ribavirin, *TPV* telaprevir, *ICC* intraclass correlation coefficient

t statistic p values: * p < 0.05; ** p < 0.01; *** p < 0.001

Eleven patients (11%) discontinued HCV therapy due to treatment-related side effects (9) or being lost to follow-up (2). A higher proportion of patients treated with IFN-containing regimens prematurely discontinued therapy due to side effects than those treated with IFN-free DAA (12.8% vs. 3.1%, P = 0.02). The two patients lost to follow-up received IFN-DAA treatment with sofosbuvir/ledipasvir. Two patients discontinued their IFN-free DAA due to worsening insomnia, gastrointestinal side effects, and severe fatigue. One of the latter two patients had a history of decompensated cirrhosis and received sofosbuvir/ledipasvir. The other patient had minimal liver fibrosis and received ombitasvir/paritaprevir/ritonavir plus dasabuvir and ribavirin. The mean and standard deviation of the total HCV-SI T-scores of the 11 patients discontinuing treatment were 55.66 and 11.96, respectively. In the HCV premature treatment discontinuation model, the predicted total HCV-SI marginal scores (fixed portion) during treatment were higher (p < 0.05) among those discontinuing early due to side effects than among those who completed treatment (Fig. 1b). Table 4 presents the mixed model results for the regression of HCV-SI T-scores on premature treatment discontinuation, demonstrating that the total HCV-SI significantly predicted those patients who prematurely discontinued HCV treatment. We observed similar effects for the somatic and neuropsychiatric subscales but not for the sleep scores.

**Table 4:** Mixed model regression: symptom (subscales) T-scores on premature HCV treatment discontinuation due to side effects or loss to follow-up

Factor (Reference: all others)	T-score, all symptoms B (95% CI)	T-score, somatic B (95% CI)	T-score, sleep B (95% CI)	T-score, neuropsyp. B (95% CI)
Non-completer, AE/side effects	7.33 (1.05, 13.61)*	7.46 (0.70, 14.22)*	3.72 (−0.67, 8.10)	6.28 (1.31, 11.24)*
Constant	48.85 (47.03, 50.68)***	48.90 (47.12, 50.68)***	49.53 (47.85, 51.20)***	49.06 (47.24, 50.87)***
No. of patients	103	103	103	103
ICC	0.6	0.59	0.57	0.58

*B* Beta coefficient, *CI* Confidence intervals, *AE* Adverse events, *ICC* intraclass correlation coefficient

t statistic p-values: * p < 0.05; *** p < 0.001

Additional file 3: Table S3 presents ROC areas adjusted for within-subject clustering by the source of variation (between- and within- subject) and by discriminating criteria for total HCV-SI and its subscales, as discussed in Methods. Discrimination was poorest for the sleep subscale and tended unsurprisingly to be higher for within-subject discrimination than between subject discrimination. For the binary regimen composition criterion, ROC area confidence intervals excluded the null value of 0.5 for the total HCV-SI and the somatic and neuropsychiatric subscales on both between and within-subject estimates. For the premature treatment discontinuation criterion, ROC area confidence intervals excluded 0.5 for all within-subject estimates but only for the neuropsychiatric subscale for between-subject estimates. Comparing the discrimination of the total HCV SI to that of the PROMIS depression score, the total HCV SI was somewhat more discriminating for both criteria, for both within and between subject estimates.

## Discussion

This study used the HCV-SI to evaluate the evolution of symptoms of PLWH before, during and after HCV treatment, and across three different HCV treatment eras. The HCV-SI discriminated among treatment regimens known to vary by their side effect profiles, and between those patients with and without premature HCV treatment discontinuation due to treatment-related side effects or loss to follow-up. These findings suggest that the HCV-SI can be used to monitor PLWH symptoms while undergoing HCV therapy.

If treating HCV with IFN-free DAA is shorter, better tolerated and more effective than the previous IFN-based therapies, why are quantitative instruments to monitor symptoms during HCV treatment with DAA of value? Improved access to contemporary HCV treatment regimens in patients with ongoing barriers to care and medical comorbidities is likely [15]. Ongoing structured screening of patient symptoms during treatment could provide counseling opportunities for symptom management and dosing recommendations (e.g. correcting the inappropriate use of antacids with sofosbuvir/ledipasvir) [16]. Use of a standardized assessment tool (such as the HCV-SI) facilitates a consistent approach for less experienced providers (such as nurse practitioners, physician assistants, and clinical pharmacists) to recognize patients at risk of treatment discontinuation [17, 18]. Hence, the HCV-SI allows standardization of patients’ self-reported safety issues. Additionally, initial licensing studies suggested that discontinuation of DAA treatment among PLWH due to side effects or lost to follow-up was exceedingly rare [19]. However, data from actual clinical care in our clinic showed that during the first 2-years of DAA use, 5% of PLWH discontinued HCV therapy due to side effects or loss to follow-up [20]. Moreover, unlike with IFN-based treatments, patients discontinuing IFN-free DAA therapy are at risk of developing HCV resistance and potential transmission of HCV resistance [21]. Finally, incorporating symptom monitoring and concurrent drug and alcohol screening during and after HCV therapy can identify behaviors that put patients at risk for HCV reinfection [22]. The value of quantitative inventories such as the one presented here to direct appropriate behavior change will be valuable in this regard [23].

The HCV-SI demonstrated discriminant validity based on HCV treatment composition, showing that patients receiving DAA had significantly lower scores than those treated with IFN-containing regimens. Consistent with results from clinical trials [24], most of our patients treated with IFN-Free DAA tolerated their regimens well, despite having a high proportion of patients with cirrhosis and even prior liver decompensation. In fact, among HCV-infected PLWH, those treated with IFN-free DAA had lower symptom scores than did untreated patients, particularly notable given that 10% of the patients treated with IFN-free DAA also received ribavirin.

Importantly, the HCV-SI effectively predicted patients who prematurely discontinued HCV therapy either due to side effects or loss to follow-up. Patients who discontinued HCV therapy due to side effects had significantly higher symptom scores than those who finished HCV therapy successfully.

This study had several limitations. First, the limited number of patients who prematurely discontinued IFN-free DAA therapy limits the ability to fully explore the value of the instrument since changes were primarily reported as averages across patient groups. Secondly, while the mixed effect models demonstrated statistical significance, the ROC areas, especially for between-subject estimates, were only moderate. Third, the sample size is relatively small, and the results may not be generalizable to other populations with different sociodemographic and clinical characteristics. Finally, although we found no major differences between those completing HCV-SI and those not completing it, it is possible that unmeasured differences existed between the included and excluded treated patients. Nevertheless, the results of this study highlight the value of incorporating quantitative tools such as the HCV-SI to measure and track subjective manifestations of PLWH to help identify those patients at risk of treatment discontinuation.

## Conclusions

The HCV-SI showed discriminant validity across different HCV regimen compositions and identified patients at risk of HCV treatment discontinuation due to adverse events. Medical providers treating HCV among PLWH could consider the HCV-SI as a patient-reported outcome.

## Additional files

**Additional file 1.** The hepatitis C symptom inventory.

**Additional file 2.** Comparison of patients who were included in (n=103) versus those excluded (n=51) from the study.

**Additional file 3.** Harrell’s c-statistics (ROC areas) by Discriminating Criteria, Source of Discrimination, HCV-SI Subscales and PROMIS Depression Score.
