# Supplementary material for: Validation of a questionnaire to monitor symptoms in HIV-infected patients during hepatitis C treatment
Source: AIDS Res Ther. 2017 Sep 20;14:56. doi: 10.1186/s12981-017-0182-7 (PMC5607579; doi:10.1186/s12981-017-0182-7)
Supplement: Supplementary file 1 — Additional file 1. The hepatitis C symptom inventory. [file 12981_2017_182_MOESM1_ESM.docx]

Supplementary Table 1:

The Hepatitis C Symptom Inventory

Symptoms vary from day to day, week to week. Please indicate the level of severity that best characterized your experience of each symptom (if any) over the past week by choosing the circle that matches your response. *This question is required.

|  | Absent | Mild | Moderate | Severe | Very Severe |
| --- | --- | --- | --- | --- | --- |
| 1. Anxiety |  |  |  |  |  |
| 2. Cough or nasal congestion |  |  |  |  |  |
| 3. Sadness/depression |  |  |  |  |  |
| 4. Restlessness |  |  |  |  |  |
| 5. No interest in activities |  |  |  |  |  |
| 6. Difficulty making decisions |  |  |  |  |  |
| 7. Strange thoughts |  |  |  |  |  |
| 8. All-over sick feeling |  |  |  |  |  |
| 9. Difficulty getting to sleep |  |  |  |  |  |
| 10. Difficulty staying asleep |  |  |  |  |  |
| 11. Sleeping too much |  |  |  |  |  |
| 12. Nausea |  |  |  |  |  |
| 13. Vomiting |  |  |  |  |  |
| 14. Loss of appetite |  |  |  |  |  |
| 15. Tiredness/fatigue |  |  |  |  |  |
| 16. Distractability |  |  |  |  |  |
| 17. Body aches |  |  |  |  |  |
| 18. Joint pain |  |  |  |  |  |
| 19. Chest pain |  |  |  |  |  |
| 20. Other pain |  |  |  |  |  |
| 21. Episodes of confusion |  |  |  |  |  |
| 22. Word finding problem |  |  |  |  |  |
| 23. Memory problem |  |  |  |  |  |
| 24. Irritability |  |  |  |  |  |
| 25. Decreased motivation |  |  |  |  |  |
| 26. Hallucinations |  |  |  |  |  |
| 27. Lack of emotions |  |  |  |  |  |
| 28. Mood swings |  |  |  |  |  |
| 29. Slowed movement |  |  |  |  |  |
| 30. Tremor/shakiness |  |  |  |  |  |
| 31. Walking problems |  |  |  |  |  |
| 32. Vision problems |  |  |  |  |  |
| 33. Bladder problems |  |  |  |  |  |
| 34. Loss of interest in sex |  |  |  |  |  |
| 35. Fever |  |  |  |  |  |
| 36. Headaches |  |  |  |  |  |
| 37. Nightmares |  |  |  |  |  |
| 38. Shortness of breath |  |  |  |  |  |
| 39. Rash/skin change/itching |  |  |  |  |  |
| 40. Change in hair |  |  |  |  |  |
| 41. Dizziness |  |  |  |  |  |

The questions classified according to each of the three subscales: Somatic, Neuropsychiatric, and Sleep symptoms are noted below:

Neuropsychiatric score: q1+q3+q4+q5+q6+q7+q14+q15+q16+q21+q22+q23+q24+q25+q28+q36+q38 (17 items)

Somatic score: q8+q12+q17+q18+q19+q20+q29+q31+q39+q40+q41 (11 items)

Sleep score: q9+q10 (2 items)
