# Supplementary material for: Validation of a questionnaire to monitor symptoms in HIV-infected patients during hepatitis C treatment
Source: AIDS Res Ther. 2017 Sep 20;14:56. doi: 10.1186/s12981-017-0182-7 (PMC5607579; doi:10.1186/s12981-017-0182-7)
Supplement: Supplementary file 2 — Additional file 2. Comparison of patients who were included in (n=103) versus those excluded (n=51) from the study. [file 12981_2017_182_MOESM2_ESM.docx]

Supplementary Table 2: Comparison of patients who were included in (n=103) versus those excluded (n=51) from the study.

| Patient characteristic | Completed the HCV-SI in two-time phases (n=103) | Decline to complete or completed only one-time phase of the HCV-SI (n = 51) | p-value |
| --- | --- | --- | --- |
| Age, median (IQR) | 51.0 (44.0, 55.0) | 53 (47-61) | 0.072 |
| Sex  Female  Male | 7 (6.8%)  96 (93.2%) | 41 (80.4%)  10 (19.6%) | 0.75 |
| Race  Non-white  White | 27 (26.2%)  76 (73.8%) | 18 (35.3%)  33 (64.7%) | 0.24 |
| Ethnicity  Not hispanic  Hispanic | 85 (82.5%)  18 (17.5%) | 42 (82.4%)  9 (17.6%) | 0.98 |
| HIV risk factor  Men who have sex with men  Heterosexual  Hemophilia  Men who have sex with men IDU  Heterosexual IDU | 24 (23.3%)  1 (1.0%)  6 (5.8%)  45 (43.7%)  27 (26.2%) | 13 (25.5%)  1 (1.9%)  2 (3.9%)  12(23.5%)  23 (45.2%) | 0.09 |
| CD4+ cells/mm^3^, median (IQR) | 476 (341, 693) | 563 (372, 784) | 0.14 |
| HIV viral load copies  ≤50  >50 | 94 (91.3%)  9 (8.7%) | 48 (94.1%)  3 (5.9% | 0.53 |
| HCV viral load in millions IU/L, median (IQR) | 2.0 (0.4, 5.8) | 2.4 (0.6, 5.4) | 0.88 |
| HCV genotype  1/1A/1B  2  3  4 | 86 (83.5%)  4 (3.8%)  11 (10.7%)  2 (1.9%) | 42 (82.3%)  2 (3.9%)  5 (9.8%)  2 (3.9%) | 0.91 |
| Prior hepatitis C treatment history  Naïve  Interferon-intolerant  Interferon-failure  Cure but reinfected | 80 (77.7%)  6 (5.8%)  16 (15.5%)  1 (1.0%) | 32 (62.7%)  5 (9.8%)  12 (23.6%)  2 (3.9%) | 0.21 |
| Cirrhosis status  Non-cirrhotic  Cirrhotic | 70 (68.0%)  33 (32.0%) | 32 (63%)  19 (37%) | 0.52 |
| Prior Decompensation or CPS > B  Compensated cirrhosis  Prior decompensated cirrhosis  Non-cirrhotic | 20 (19.4%)  13 (12.6%)  70 (68.0%) | 16 (31.4)  3 (5.8)  32 (62.8) | 0.16 |
| SVR on most recent treatment  Yes  No, viral failure  No, sides effects/adverse event  No, died during treatment | 78 (76.5%)  11 (10.8%)  9 (8.8%)  4 (3.9%) | 48 (94%)  3 (6.0%)  0  0 | 0.034 |
| Barriers to care  Ongoing alcohol/drug use  Active psychiatric disease  Unstable housing | 29 (28.2%)  27 (26.2%)  5 (4.9%) | 15 (29.4%)  17 (33.3%)  3 (5.9%) | 0.87  0.36  0.79 |

HCV-SI = hepatitis symptom inventory; IQR = interquartile range; IDU = intravenous drug use; CPS = Child-Pugh score
