# Supplementary material for: Validation of a questionnaire to monitor symptoms in HIV-infected patients during hepatitis C treatment
Source: AIDS Res Ther. 2017 Sep 20;14:56. doi: 10.1186/s12981-017-0182-7 (PMC5607579; doi:10.1186/s12981-017-0182-7)
Supplement: Supplementary file 3 — Additional file 3. Harrell’s c-statistics (ROC areas) by Discriminating Criteria, Source of Discrimination, HCV-SI Subscales and PROMIS Depression Score. [file 12981_2017_182_MOESM3_ESM.docx]

Supplementary Table 3: Harrell's c-statistics (ROC areas) by Discriminating Criteria, Source of Discrimination, HCV-SI Subscales and PROMIS Depression Score.

| Criteria: | Pegylated interferon/ribavirin +/- telaprevir  [reference: DAA+ no treatment] | | Premature Treatment Discontinuation  [reference: completed] | |
| --- | --- | --- | --- | --- |
| Source | Between | Within | Between | Within |
| T-score All Symptoms | 0.69 (0.59,0.78) | 0.78 (0.67,0.90) | 0.57 (0.40, 0.75) | 0.91 (0.86, 0.95) |
|  |  |  |  |  |
| T-score Somatic | 0.66 (0.56, 0.76) | 0.92 (0.84,0.99) | 0.60 (0.44, 0.76) | 0.96(0.94, 0.98) |
|  |  |  |  |  |
| T-Score Sleep | 0.55 (0.45, 0.66) | 0.45 (0.20, 0.70) | 0.49 (0.30, 0.68) | 0.67 (0.59, 0.75) |
|  |  |  |  |  |
| T-Score Neuropsychiatric | 0.67 (0.58, 0.77) | 0.69 (0.56, 0.81) | 0.57 (0.56, 0.81) | 0.86 (0.79, 0.93) |
| Depression T-score | 0.63 (0.53, 0.73) | 0.73 (0.55, 0.91) | 0.53 (0.38, 0.68) | 0.83 (0.75, 0.91) |

ROC = receiver operating characteristics; HCV-SI= hepatitis C symptoms inventory; DAA= direct acting antivirals

95% confidence intervals in parentheses.
